# Using Micromechanical Resonators to Measure Rheological Properties and Alcohol Content of Model Solutions and Commercial Beverages

**DOI:** 10.3390/s120506497

**Published:** 2012-05-16

**Authors:** Rosemary Paxman, Jake Stinson, Anna Dejardin, Rachel A. McKendry, Bart W. Hoogenboom

**Affiliations:** London Centre for Nanotechnology, Departments of Physics, Chemistry, and Medicine, University College London, 17-19 Gordon Street, London WC1H 0AH, UK; E-Mails: r.paxman@ucl.ac.uk (R.P.); j.stinson@ucl.ac.uk (J.S.); anna.dejardin@ucl.ac.uk (A.D.); r.a.mckendry@ucl.ac.uk (R.A.M.)

**Keywords:** micromechancical sensors, cantilevers, rheological properties, alcohol contents, quality control

## Abstract

Micromechanic resonators provide a small-volume and potentially high-throughput method to determine rheological properties of fluids. Here we explore the accuracy in measuring mass density and viscosity of ethanol-water and glycerol-water model solutions, using a simple and easily implemented model to deduce the hydrodynamic effects on resonating cantilevers of various length-to-width aspect ratios. We next show that these measurements can be extended to determine the alcohol percentage of both model solutions and commercial beverages such as beer, wine and liquor. This demonstrates how micromechanical resonators can be used for quality control of every-day drinks.

## 1. Introduction

The dynamic characteristics of cantilever beams strongly depend on the rheological properties of the fluid in which the beams are immersed. Initial investigation into such fluid effects, using millimeter-sized cantilevers, dates back to the 1960s [1]. Following the advent of atomic force microscopy (AFM) twenty years later [2] and the resulting increase in microcantilever production, these investigations were extended to micro-scale sensors. The resonant behaviour of such microcantilevers is directly related to the fluid viscosity and density, a property which has been used in the measurement of rheological properties [3–11]. Microcantilever or microresonator devices offer the advantage of fast, miniaturized and localized monitoring, using only μL sample requirements, thus providing a valuable means of fluid control whilst also helping to overcome existing measurement problems such as blockages, time consuming calibration processes, expensive equipment costs and sensitivity to vibrations [12–14].

Studies into the density and viscosity of petroleum and silicon oils have demonstrated the commercial potential of micromechanical resonators for rheological measurements [3,15–18]. ‘*In-situ*’ fluid experiments [16,17] using singly-clamped devices have successfully measured the density and viscosity of petroleum fluids [17], with results lying within a ±0.35% and ±3% degree of uncertainty, respectively. Micromechanical resonators have also been used to measure the density and viscosity of glycerol and ethanol solutions [3,5], resulting, e.g., in a measured viscosity of (1.05 ± 0.31) × 10^−3^ Pa·s for ultrapure ethanol (compared to the expected 1.35 × 10^−3^ Pa·s) [3], using Sader's model [19] to relate the cantilever resonance frequency and quality factor to rheological properties.

Biological applications of nanomechanical rheological sensors have also been investigated, with the characterization of sugar solutions [4] and DNA hydrolysis [5]. Hennemeyer [4] successfully monitored the change in cantilever resonant frequency and quality factor as a function of increasing sucrose, fructose and glucose solution at biologically relevant concentration, with viscosities determined within an error of less than 5%. Further biological utility has been demonstrated [5], where changes in viscosity upon the hydrolysis of double stranded DNA by DNase I was successful followed using cantilevers.

One as yet unexplored application of micromechanical resonators is the measurement of ethanol concentration, density and viscosity of alcoholic beverages. Measurements of the ethanol content, density and viscosity of alcoholic drinks are essential for analysis and quality control procedures. Many techniques are currently used in beverage analysis [20,21], with the requirements associated with real-time, industrial rheological characterization of fluids being numerous and complex [21]. These include exposure to aggressive process conditions and high cleanliness requirements. Ideally, sensors should have minimal possibility of fouling and be easily cleaned *in-situ*. In addition, they should offer a fast readout and require a low sample volume [21], which is a particular attractiveness of using micromechanical resonators.

Previous studies have investigated to what extent fluid properties and cantilever-length–to-width ratio (aspect ratio) influence the rheological calculations [7,19]. Sader [19] presented the first general theoretical model of the cantilever resonance frequency for a beam of arbitrary cross section, immersed in fluid and excited by an arbitrary driving force. Unlike previous formulations, this model quantitatively accounts for cantilever geometry and additional fluid loading, therefore allowing the frequency response of the beam to be determined based on cantilevers properties and the fluid viscosity and density alone. A key assumption in this model is that the length of the beam must greatly exceed its width, *i.e.*, it has a high aspect ratio. Chon [7] examined this model using a range of cantilevers, each of varying dimensions, immersed in acetone, water, CCl_4_ and 1-butanol. They found the model to correctly reproduce the frequency response of the cantilevers within an error of 10% for aspect ratio ranges of *L*/*w* = 4 – 14.

Here we focus on the accuracy of such measurements to determine mass density and viscosity of fluids, and in particular to their application for the characterization of commercial beverages. In that context, one of the key parameters is the alcohol percentage. We first compare different aspect ratios of rectangular cantilevers that are clamped at one end, using identical solutions and experimental set-up, to examine the extent by which aspect ratio influences the measurement of density and viscosity. To demonstrate their potential for real-time drinks analysis, we present density and viscosity measurements on a range of commercial drinks, comparing our results to simple aqueous ethanol solutions. We then use these data to determine alcohol content, comparing it to the specifications by the manufacturers. We also investigate the validity of current theory on more viscous liquids, using aqueous glycerol solutions at differing concentrations.

## 2. Methods

**Figure 1.**
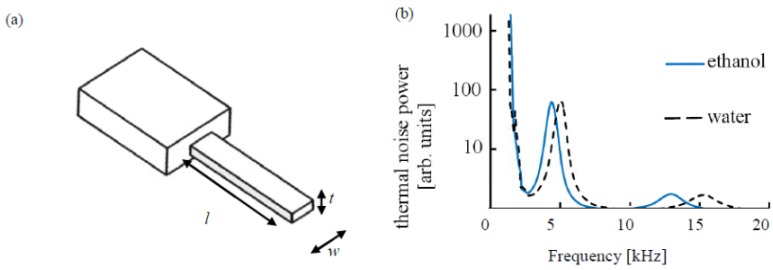
(**a**) Schematic of the cantilevers used in fluid measurements. (**b**) Thermal noise power spectrum for a cantilever of geometry 500 × 100 × 0.9 μm^3^, oscillating in water (black, dashed) and ethanol (blue), plotted against a logarithmic scale. The second and third oscillatory modes are shown, with *f^(n)^_med_* and *Q^(n)^_med_* typically between 4–5 kHz and 1.5–2 for the second mode and 12–15 kHz and 2.5–3 for the third mode, respectively.

We consider singly clamped rectangular cantilevers of length, *l*, width, *w*, and thickness *t*, oscillating in a viscous medium. The cantilever design and a typical thermal noise measurement are illustrated in Figure 1.

The relevant mathematical framework for describing the resonance behavior of a cantilever with length greatly exceeding its width, and width greatly exceeding its thickness, has been developed by others [19]. It is important to note that the model used is strictly valid only for Q ≫ 1. For this reason, we do not take the fundamental mode into consideration, as for the cantilevers used here, its Q ≈ 1. Here we give the main two relevant equations for convenience, while referring to [19] for the explicit form of the used hydrodynamic function Γ(*w,f*^(^*^n^*^)^*_med_, ρ_med_, η*).

When oscillated in a liquid of mass density *ρ_m_* and viscosity *η*, the natural frequency, *f^(n)^_med_*, of *n*-th mode of flexural cantilever oscillation is given by:

(1) $${f^{\left(n\right)}}_{\text{med}}={f^{\left(n\right)}}_{\text{vac}}{\left(1+\frac{\pi \rho_{m}w}{4\rho_{c}t}\Gamma_{r}(w,{f^{\left(n\right)}}_{\text{med}},\rho_{\text{med}},\eta )\right)}^{-1/2}$$

where *f^(n)^_vac_* is the resonance frequency of the cantilever in vacuum, *ρ_c_* is its mass density and Γ is the hydrodynamic function for a rectangular beam [19]. This describes the hydrodynamic loading experienced by the cantilever. Subscripts *r* and *i* are used to denote the real and imaginary parts of Γ, respectively. In its simplest form, Γ depends on cantilever width, *f^(n)^_med_* and the density and viscosity of the medium [19]. We note that there are more complex formulations available [22], extending [19] to 3-D, accounting for increasing mode numbers. In the form used here [19], its accuracy decreases as the mode number increases [22]. The extended model also includes a formulation for torsional modes, which typically exhibit a higher Q. Here we restrict our analysis to the earlier model, since—if sufficiently accurate—it provides a solution with the advantage of simple and straightforward numerical implementation.

According to [19] the effects of viscous damping on the quality factor are:

(2) $${Q^{\left(n\right)}}_{\text{med}}=\frac{\frac{4\rho_{c}t}{\pi \rho_{m}w}+\Gamma_{r}(w,{f^{\left(n\right)}}_{\text{med}},\rho_{m},\eta )}{\Gamma_{i}(w,{f^{\left(n\right)}}_{\text{med}},\rho_{m},\eta )}$$

Given the relative simplicity of Γ [19], this set of two implicit equations can be solved numerically to yield the mass density and viscosity of the medium. This is executed using the numerical nonlinear least squares regression algorithm in Mathematica (Wolfram Wolfram Research Inc., Champaign, IL, USA). Calibration of the cantilever is needed to obtain the resonance frequency in vacuum *f^(n)^_vac_* and cantilever thickness *t* with sufficient accuracy in Equations (1) and (2) [23]. This procedure was outlined by Boskovic [8], with air used as the reference medium of known density and viscosity, as done in the measurements presented in this work. When the inertial effects of the fluid are small compared to its dissipative effects (low Reynolds number), such as for soft cantilevers in aqueous solutions, we find this procedure to be less accurate.

Once the mass density and viscosity of the medium have been determined, model solution data can be mapped on the literature values for aqueous ethanol solutions [24] to find the ethanol (alcohol) percentage. We test this procedure on pure aqueous ethanol solutions and on a range of beverages, of varying alcohol content. The beverages are measured as purchased, without any further treatment.

For our experiments, the cantilever is mounted in a commercial atomic force microscope (Nanowizard I, JPK Instruments, Berlin, Germany) and submerged in 100–200 μL of liquid, where it is subject to thermal fluctuations, which is the only actuation present in these measurements. Optical beam-deflection is employed to monitor cantilever deflections, owing to its relative simplicity and high lateral resolution. A laser is focused on the free end of the cantilever and reflected back onto the Position Sensitive Detector (PSD). Cantilever motion will consequently change the laser position on the photodiode and therefore the light intensity on each cell. Prior to measurement, the laser spot is aligned in the centre of the PSD, so each segment has equal levels of illumination. Ethanol (ACS reagent, ≥99.5%) and glycerol (ACS reagent, ≥99.5%) used to make the required solutions were obtained from Sigma Aldrich (St. Louis, MO, USA). The resonance behavior of the cantilever is determined from the digitally calculated and averaged Fourier spectrum of the thermal noise of the cantilever. The thermal noise spectrum is then fitted with a simple harmonic oscillator model, via which *f^(n)^* and *Q^(n)^* are determined. Because of the broadness of the peaks, different modes of oscillation are not strictly separated any more in the frequency domain. To enhance the accuracy of the fitting procedure, we subsequently fit the different modes (up to *n* = 3) in the thermal noise spectrum, starting with the first (fundamental) mode, and for each higher mode fit the thermal noise spectrum after subtracting the fit(s) of the lower mode(s) from the experimental data. This prevents the tails of the lower modes from polluting the fits to the higher modes of oscillation. The fitting procedure is implemented in Mathematica. The measurements are carried out at a temperature of 20.5 °C.

We use tipless uncoated single-crystal silicon cantilevers with dimensions: 500 × 100 × 0.9 μm^3^, aspect ratio 1:5 [25] (IBM); 350 × 35 × 1 μm^3^, aspect ratio 1:10 (NSC12 tipless cantilevers, MikroMasch, Tallin, Estonia); and 400 × 30 × 2 μm^3^, aspect ratio ∼1:13 (CLFC-NOBO tipless cantilevers, Bruker Probes, Santa Barbara, CA, USA).

We examine the applicability of the described method in determining the density and viscosity of aqueous ethanol and glycerol solutions, alcoholic drinks and also their alcohol percentage. We also examine the influence of differing aspect ratios in the accuracy of the calculation. Success of this method in determining gas properties has been previously reported [8] so we will concentrate on measurements in liquid only. Error bars show the standard deviation between repeat experiments (using different cantilevers of the same type) throughout. Typically, there were five repeats for each condition.

## 3. Results & Discussion

**Figure 2.**
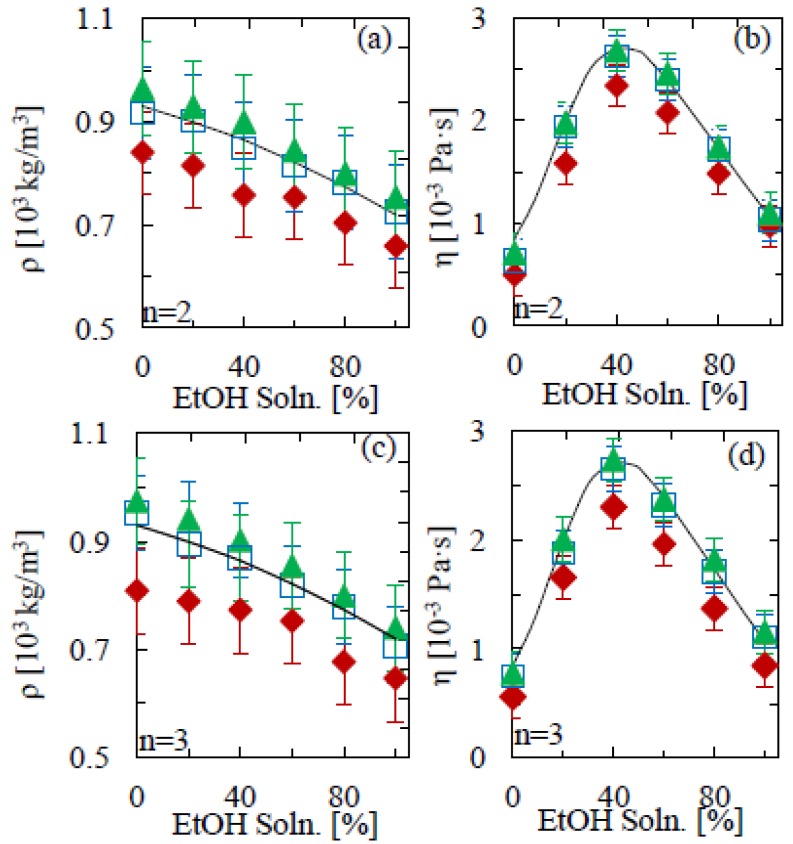
(**a**) Mass density and (**b**) viscosity of water-ethanol mixtures, as a function of ethanol volume percentage, determined from the second mode (n = 2) of flexural cantilever oscillation. (**c**) and (**d**) show the corresponding results for the third mode (n = 3) of oscillation. The solid line denotes the literature values [24] at 20 °C. Triangles, squares and diamonds denote aspect ratios of 13:1, 10:1 and 5:1, respectively.

Figure 2 shows the density and viscosity results for the second and third mode of oscillation in aqueous ethanol solutions of 0% (milliQ water), 20%, 40%, 60%, 80% and 100%. Whereas the density simply decreases as the EtOH percent increases, the pattern is less straight forward in the case of viscosity. At first we see an increase up to around 40% EtOH(aq) solution concentration, followed by a reduction between 40% and 100% EtOH(aq), with experimental data mimicking this pattern. Studies into the non-ideal mixing behavior of these solutions can be found in the literature [26,27]. Our results show identical trends to those expected from literature values [24], validating the model for detecting changes in fluid properties. As outlined by Sader [19] the length of the beam must greatly exceed its nominal width, an approximation implemented in the theoretical model. It is therefore not surprising that the success of the calculation reduces as the aspect ratio reduces, as seen in the measurements. Only for aspect ratios ≥10 do the measured data correspond to the literature values within the experimental accuracy. From Figure 2, it can be seen that the low mode numbers used here (*n* = 2 and *n* = 3) yield equivalent accuracy.

**Figure 3.**
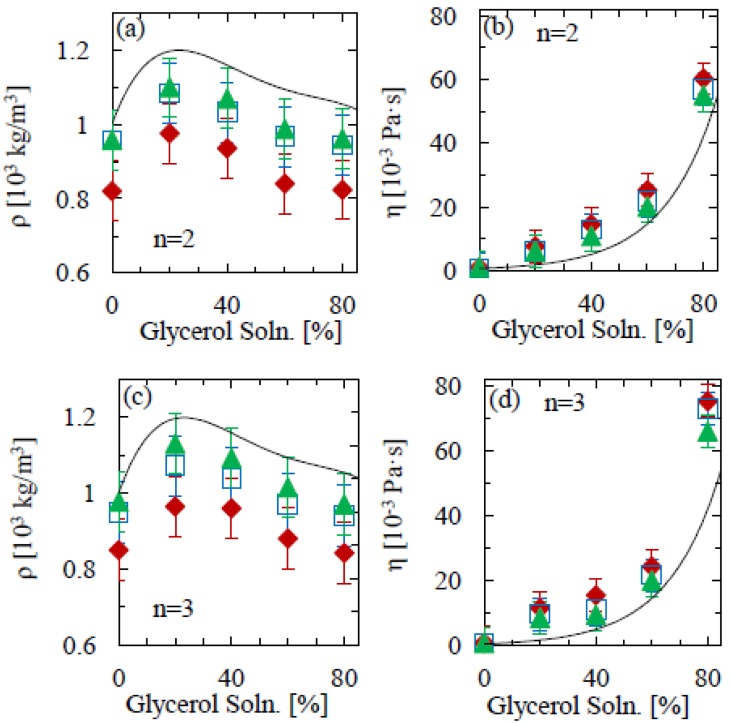
(**a**) Mass density and (**b**) viscosity of water-glycerol mixtures, as a function of glycerol volume percentage, determined from the second mode (n = 2) of flexural cantilever oscillation. (**c**) and (**d**) show the corresponding results for the third mode (n = 3) of oscillation. The solid line denotes the literature values [24] at 20 °C. Triangles, squares and diamonds denote aspect ratios of: 13:1, 10:1 and 5:1 accordingly.

We repeat the procedure for glycerol solutions of the same percentage concentration up to 80%, the results of which are shown in Figure 3.

Above 80% concentration, the solution was too viscous for any resonance behavior to be observed in the thermal noise. Again, the cantilever measurements reproduce the trend expected from the literature values, and—in agreement with previous findings [8,19]—the lower aspect ratio beam again yields the poorest match. Overall, only for the highest aspect ratio (13:1) do the experimental values and literature values show reasonable agreement within the experimental uncertainty. Interestingly, this agreement is still good for viscosities up to 0.02 g cm^−1^ s^−1^ at 60% glycerol concentration, indicating that—for high aspect ratios—the procedure is reasonably accurate up to the point where viscous damping completely suppresses the resonant behavior of the cantilever. Nevertheless, the larger viscosity implies a greater infringence on the assumption *Q* ≫ 1 in our analysis, which may be the reason for the slightly larger deviation of measured mass densities and viscosities from the literature values, as compared to the water-ethanol results.

We next apply the procedure to the analysis of commercially available non-alcoholic and alcoholic beverages. As previous results have demonstrated the importance of a high (length-to-width) aspect ratio for the cantilever, we only use cantilevers where the length-to-width ratio is 10:1. For smaller aspect ratios, measurements for both mass density and viscosity will become increasingly inaccurate, as can be observed in Figure 2.

**Figure 4.**
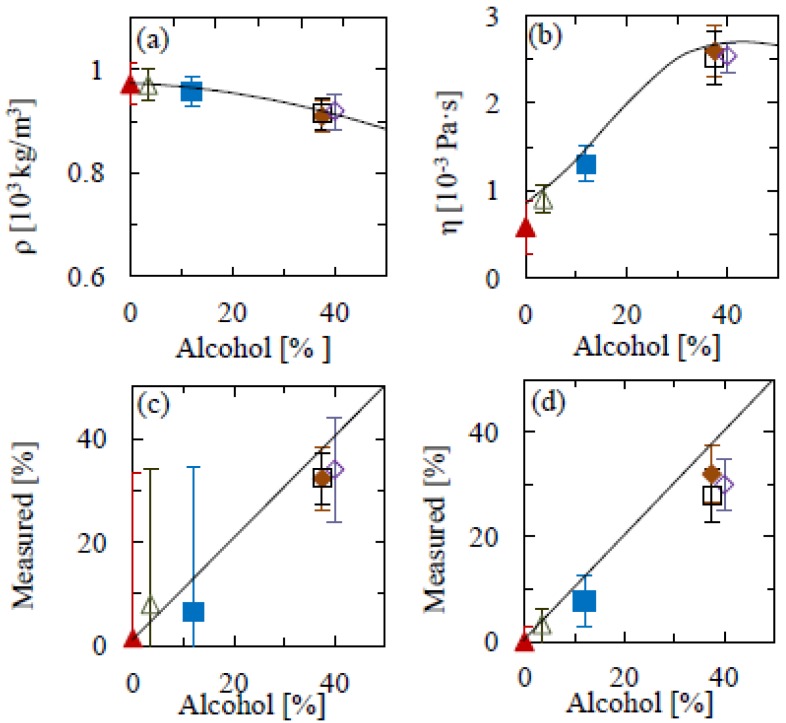
(**a**) Measured mass density and (**b**) viscosity of commercial beverages as a function of specified alcohol content. (**c**) and (**d**): Alcohol percentage determined from the measured mass density (**c**) and viscosity (**d**) as a function of specified alcohol content. Symbols: triangle—non-alcoholic beer; open triangle—3.5% beer; square—12.5% white wine; open square—37.5% gin; diamond—37.5% vodka; open diamond—40% whisky. Solid lined denotes interpolated water/ethanol data from Figure 2.

Figure 4 shows density and viscosity measurements for a range of drinks. These are: beer, non-alcoholic beer, white wine, vodka, whisky and gin. We then use these values to determine the alcohol content. Upon application to alcoholic drinks, we find a good agreement between experimental and interpolated water/ethanol solution values for density and viscosity. By comparing the thus determined rheological properties to values for ideal water-ethanol solutions as measured previously, we determine the alcohol percentage in the beverages, and find these in good agreement with the manufacturers' specifications (Figure 3). The alcohol content could be determined from the mass density and from the viscosity separately, though the uncertainties are such that the viscosity is a far more accurate indicator of alcohol content for these beverages. Deviations from the expected values could be due to the interplay of other components within the drink, for example glycerol content, of which ranges from 5–7 g/L in wine [28], 0.9–2 g/L in beer [29] and higher in spirits, where the alcohol percentage is greater.

The accuracy of the technique could be further improved by using cantilevers of a higher aspect ratio. Figures 2 and 3 illustrate the importance of this, showing a clear variation across geometries. The differences between experimental mass densities and literature values is on average 16% for 5:1 aspect ratio cantilevers; however this is significantly reduced to 4% for the longer beams. A similar scenario is seen with the viscosity measurements, where the differences between literature and experimental values for the 5:1 and 13:1 aspect ratios are 25% and 6%, respectively. On the other hand, the results in Figure 4 show that such deviations can be avoided by calibrating the mass density and viscosity measurements in model solutions. The main uncertainty would thus be in variations in the curve fitting, temperature and cantilever geometry, for all of which there is large scope for improvement by standardization and repeat measurements, as well as by fitting both amplitude and phase of the resonance response to external actuation (as opposed to thermal fluctuations).

The main scope for improvement in the described technique lies within the readout method. Here cantilever deflection is measured via an optical readout system, limiting the technique to transparent liquids and also reducing its use as a portable device. This could be overcome via the use of piezoelectric or piezoresistive cantilevers, or other schemes that bypass the need of optical detection.

## 4. Summary and Conclusions

In summary, we have experimentally demonstrated the use of micromechanical cantilever sensors to extract the density and viscosity of simple binary laboratory solutions and of more complex commercial drinks. We have also demonstrated that alcohol content in beverages can be determined from such measurements. As expected, the accuracy of the measurements decreases with decreasing (length to width) aspect ratio of the cantilever. This is important when considering industrial applications, where margin for error is minimal.
